# Plasma free fatty acid levels influence Zn^2+^-dependent histidine-rich glycoprotein–heparin interactions via an allosteric switch on serum albumin

**DOI:** 10.1111/jth.12771

**Published:** 2014-11-22

**Authors:** O Kassaar, U Schwarz-Linek, C A Blindauer, A J Stewart

**Affiliations:** * School of Medicine, University of St AndrewsSt Andrews, UK; † Biomedical Sciences Research Complex, University of St AndrewsSt Andrews, UK; ‡ Department of Chemistry, University of WarwickCoventry, UK

**Keywords:** fatty acids, heparin, histidine-rich glycoprotein, plasma albumin, zinc

## Abstract

**Background:**

Histidine-rich glycoprotein (HRG) regulates coagulation through its ability to bind and neutralize heparins. HRG associates with Zn^2+^ to stimulate HRG–heparin complex formation. Under normal conditions, the majority of plasma Zn^2+^ associates with human serum albumin (HSA). However, free fatty acids (FFAs) allosterically disrupt Zn^2+^ binding to HSA. Thus, high levels of circulating FFAs, as are associated with diabetes, obesity, and cancer, may increase the proportion of plasma Zn^2+^ associated with HRG, contributing to an increased risk of thrombotic disease.

**Objectives:**

To characterize Zn^2+^ binding by HRG, examine the influence that FFAs have on Zn^2+^ binding by HSA, and establish whether FFA-mediated displacement of Zn^2+^ from HSA may influence HRG–heparin complex formation.

**Methods:**

Zn^2+^ binding to HRG and to HSA in the presence of different FFA (myristate) concentrations were examined by isothermal titration calorimetry (ITC) and the formation of HRG–heparin complexes in the presence of different Zn^2+^ concentrations by both ITC and ELISA.

**Results and conclusions:**

We found that HRG possesses 10 Zn^2+^ sites (*K*′ = 1.63 × 10^5^) and that cumulative binding of FFA to HSA perturbed its ability to bind Zn^2+^. Also Zn^2+^ binding was shown to increase the affinity with which HRG interacts with unfractionated heparins, but had no effect on its interaction with low molecular weight heparin (˜ 6850 Da). [Correction added on 1 December 2014, after first online publication: In the preceding sentence, “6850 kDa” was corrected to “6850 Da”.] Speciation modeling of plasma Zn^2+^ based on the data obtained suggests that FFA-mediated displacement of Zn^2+^ from serum albumin would be likely to contribute to the development of thrombotic complications in individuals with high plasma FFA levels.

## Introduction

Histidine-rich glycoprotein (HRG) is a plasma adaptor protein present at a concentration of 1.3–2.0 μm in adult blood [1,2]. HRG natively exists as a dimer, forming multiprotein complexes that regulate coagulation and other biological processes, including immune complex clearance, cell proliferation, cell adhesion, and angiogenesis [1]. This has led to its description as ‘the Swiss army knife of mammalian plasma’ [3]. High levels of HRG are associated with the clinical presentation of cardiovascular disorders, including blood vessel occlusion and thrombophilia [4–6]. HRG thus seems to play a particularly important role in regulating blood clotting. The primary structure of HRG contains two cystatin-like domains at the N-terminus, a histidine-rich region (HRR) flanked by two proline-rich regions, and a C-terminal domain [7,8]. The distinctive HRR is composed of repeating GHHPH motifs [1]. This domain associates with Zn^2+^ to alter the binding characteristics of the protein, such that the affinity of HRG for a number of molecules, including the natural anticoagulants heparin and heparan sulfate, is increased [9]. This, in turn, enables neutralization of these anticoagulants, leading to a prothrombotic effect via inhibition of antithrombin III activity [10,11]. Thus, Zn^2+^ binding by HRG provides a potential means of regulating its function. An anticoagulatory role for Zn^2+^–HRG has also been suggested, as Zn^2+^ can potentiate the binding of HRG to factor XIIa [12], but this is less clear.

Indeed, plasma Zn^2+^ has emerged as an important regulator of hemostasis and thrombosis [13]. Zinc deficiency is associated with defects in platelet aggregation and increased bleeding times, effects that can be reversed with zinc supplementation [14–17]. Plasma Zn^2+^ levels are highly regulated, and under normal conditions ˜ 75% of the total 20 μm plasma Zn^2+^ (˜ 15 μm) is bound to serum albumin [18], and not to HRG [19]. Much of the remaining 5–6 μm Zn^2+^ in plasma is strongly bound to other proteins (such as α_2_-macroglobulin), with the concentration of free/exchangeable (weakly bound) Zn^2+^ in plasma thought to be in the nanomolar range [20,21]. It is thought that Zn^2+^ release from platelet-derived α-granules may provide enough Zn^2+^ locally to modify HRG–heparin interactions and aid in the initiation of coagulation [11,22]. Mahdi *et al*. [23] reported that the free Zn^2+^ concentration close to activated platelets is 7–10 μm, and may be even higher in the growing thrombus. Despite this, the Zn^2+^-binding properties of HRG and the role that Zn^2+^ plays in influencing HRG–heparin interactions are not fully understood.

**Fig 1:**
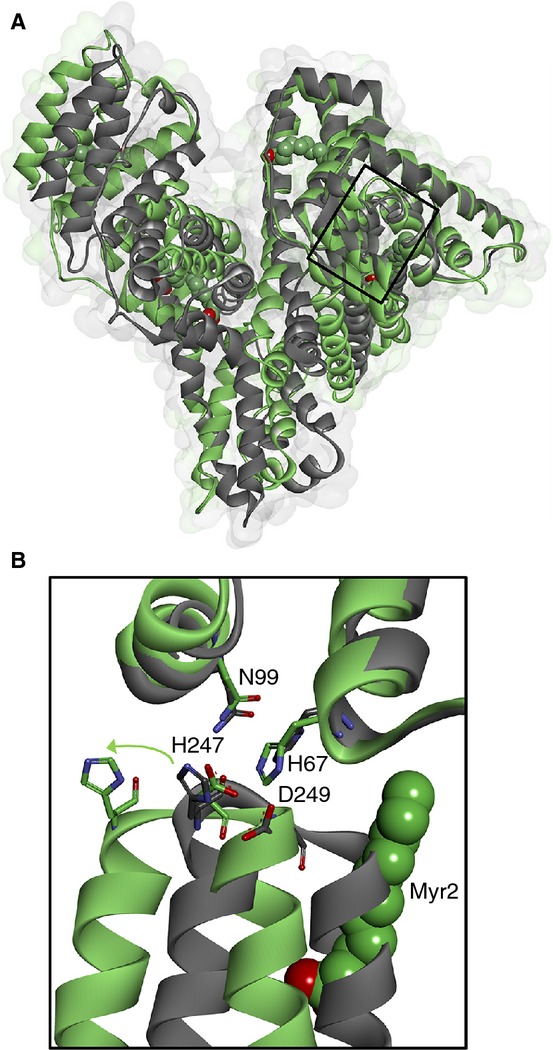
The fatty acid/Zn^2+^ switch on serum albumin. (A) Overlay of crystal structures of human serum albumin with (gray; Protein Data Bank [PDB] 1BJ5 [26]) and without (green; PDB 1AO6 [61]) myristate (Myr) bound, showing the location of the major Zn^2+^-binding site. (B) Close-up showing the movement of Zn^2+^-coordinating residues His247 and Asp249 relative to His67 and Asn99 between the two structures.

Previously, we identified the primary Zn^2+^-binding site on serum albumin (often referred to as site A), which consists of N-ligands from His67 and His247 and O-ligands from Asn99, Asp249, and H_2_O [24,25]. Serum albumin transports fatty acids in the circulation, and binds non-esterified fatty acids [termed free fatty acids [FFAs]) of various chain lengths, ranging from C10 to C24, at five high-affinity sites (termed FA1–FA5) and several lower-affinity sites [26–28]. Fatty acid binding at site FA2 induces a conformational switch that disengages the Zn^2+^-binding residues in domain II relative to those in domain I [24,29], as shown in Fig. 1. Under normal physiologic conditions, the plasma concentration of FFAs is ˜ 250–500 μm at rest [30]. This represents < 1 mole equivalent (mol eq.) relative to the plasma concentration of serum albumin. However, FFA levels are dynamic and, for instance, rise following meals and during periods of exercise. Crucially, elevated FFA levels are also associated with a range of disorders, including obesity [31,32], diabetes [33], fatty liver disease [34], and cancer [35]. For example, in obese individuals, plasma concentrations of FFA (at rest) are often two to three times higher [32], and in some cancer patients they are four to six times higher, than in controls [36]. Such disorders are associated with an increased risk of thrombotic complications [37,38]. For example, thromboembolism (caused by obstructive blood clots) is the second leading cause of death associated with malignancy [37]. Collectively, these observations led us to hypothesize that, under conditions where FFA levels are elevated, Zn^2+^ displaced from serum albumin could bind HRG to enhance its interaction with heparin/heparan sulfate and induce a procoagulatory effect [39].

With this in mind, we sought to gain a fuller understanding of the Zn^2+^-binding properties of HRG and the role of Zn^2+^ in controlling HRG–heparin interactions by using isothermal titration calorimetry (ITC) and an ELISA-based method. Furthermore, we used ITC to examine whether plasma FFA levels may regulate the Zn^2+^-dependent HRG–heparin interactions (via Zn^2+^ displacement from serum albumin) to probe the interactive binding of myristate (Myr) and Zn^2+^ to serum albumin. Myr was used because it balances solubility issues with an ability to still bind to serum albumin in a manner that closely matches that of the more physiologically relevant palmitate (C16) and stearate (C18) [26], albeit with slightly weaker affinity [40]. Zn^2+^-speciation modeling based on the resultant data suggests that the maintenance of FFA levels and/or free/exchangeable plasma Zn^2+^ levels would probably provide new avenues for therapeutic intervention in managing thrombotic complications in high-risk individuals.

## Materials and methods

### Purification of human and rabbit HRG

HRG was purified directly from either human plasma (TCS Biosciences, Buckingham, UK) or, for experiments detailed in the Supporting information, rabbit serum (Sigma-Aldrich, Poole, UK) with immobilized metal affinity chromatography. Plasma or serum was centrifuged (4000 × *g*, 30 min) and filtered through a 0.45-μm syringe filter (Sartorius, Epsom, UK), and imidazole was added (5 mm final) together with the equilibration buffer (10 mm Tris, 150 mm NaCl, 5 mm imidazole, pH 8). A 5-mL HisTrap nickel column (GE Healthcare Life Sciences, Little Chalfont, UK) was equilibrated with 5–10 column volumes of the equilibration buffer, and sample (50 mL) was loaded. The column was washed with equilibration buffer and then with a 70 : 30 mixture of equilibration/elution buffer (10 mm Tris, 150 mm NaCl, 400 mm imidazole, pH 8). HRG was eluted with elution buffer. The purified HRG sample was then dialyzed to remove any bound metals in the buffer of choice for further experiments, or in 50 mm ammonium carbonate prior to lyophilization.

### ITC

ITC experiments were carried out with a MicroCal VP-ITC instrument (GE Healthcare Life Sciences) in 50 mm Tris and 140 mm NaCl (pH 7.4) at 25 °C. Titrants (ZnCl_2_ and heparins) were added to the reaction buffer, and the pH was adjusted to 7.4 to match the buffer in the ITC cell containing the protein. Solutions were degassed at 22 °C for 15 min prior to performance of the experiment. Typical titrations performed were one 2-μL injection over 4 s followed by up to 55 injections of 5 μL over 10 s with an adequate interval of 240 s between injections to allow complete equilibration. The stirring speed was 307 r.p.m. Heats of dilution were accounted for with blank titrations performed by injecting ligand solution into reaction buffer and subtracting the averaged heat of dilution from the main experiments. Alternatively, in cases of saturated binding, blank titrations were omitted where the averaged residual signal of the last injections was used to determine the heat of dilution. Raw data were processed with microcal origin software, and data were fitted by use of the same software; the results presented are representative of multiple experiments. In all cases, the errors stated represent the fitting errors from individual experiments.

For fitting of the human serum albumin (HSA)–Zn^2+^ titration data in the presence and absence of Myr, initial values for *K1*_ITC_ and Δ*H1* for the high-affinity site A were determined with a sequential binding site model. Subsequent fits to determine site A occupancy used a ‘two sets of sites’ model, with *K1*_ITC_ and Δ*H1* fixed, and *N1* varied. Simultaneous variation of *N2*,*K2* and Δ*H2* yielded good fits, but physically unreasonable data for the latter values (but still resulted in a decrease in site A occupancy). Hence, fits with either *K2* and Δ*H2* fixed at values derived from fitting the data in the absence of Myr, or fits with *N2* fixed at either 1 or 2, were explored (Tables S1 and S2). The resulting values for *N1* from the various fits were averaged.

### ELISA

An ELISA experimental set-up was devised to investigate the interaction between HRG and heparin compounds. Unfractionated porcine plasma heparin (Acros Organics, Loughborough, UK) or low molecular weight heparin (LMWH) (6850 Da; Iduron, Manchester, UK) were coated overnight at room temperature onto a heparin-binding plate (Iduron) at a concentration of 25 μg mL^−1^ in 50 mm HEPES, 150 mm NaCl and 0.2% Tween-20 at pH 7.4. The wells were washed with the same buffer, and then blocked with the same buffer supplemented with 0.2% gelatin from fish skin (Sigma-Aldrich) for 1 h at 37 °C. Human HRG was then incubated for 2 h over a range of concentrations (0–3 μm) at 37 °C with or without ZnCl_2_. The reaction was detected with primary rabbit anti-HRG (Sigma-Aldrich) followed by alkaline phosphatase-linked anti-rabbit antibody (Sigma-Aldrich), and observed with *p*-nitrophenol phosphate substrate (Sigma-Aldrich) at 405 nm.

### Speciation modeling

The ‘Species’ module of the IUPAC Stability Constants Database (version 5.6) was employed for speciation modeling, with the conditional stability constants for HRG and HSA determined in this work, and typical physiologic concentrations for exchangeable Zn^2+^ (15 μm), HSA (620 μm), and HRG (1 or 2 μm). For the last of these, the binding site concentration was assumed to be 10 times that of HRG, according to the stoichiometry determined at high ionic strength. Averages and errors were calculated by employing various combinations of values for *N1*, *N2* and *K2* for HSA corresponding to the fitting values (Tables S1 and S2), and two different concentrations for HRG (Table S3).

## Results and discussion

**Fig 2:**
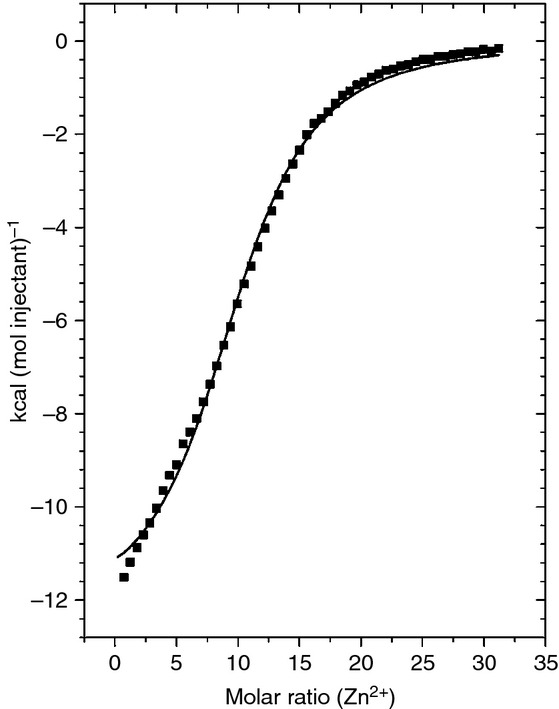
Isothermal titration calorimetry data for Zn^2+^ binding to human histidine-rich glycoprotein (HRG). Here, 55 injections of 5 μL of 150 μm ZnCl_2_ were delivered to samples of 10 μm HRG in buffer containing 50 mm Tris and 140 mm NaCl (pH 7.4) over a period of 10 s with an adequate interval (240 s) between injections to allow complete equilibration. The total Zn^2+^ concentration at the end of the experiment was 24.6 μm. Raw data are shown in Fig. S5.

We used ITC to examine the Zn^2+^-binding properties of human HRG purified from blood plasma. Zn^2+^ binding to human HRG was exothermic, and data analysis revealed that human HRG is capable of binding 10 mol eq. (*N* = 10.3) of Zn^2+^ at near-physiologic ionic strength (50 mm Tris, 140 mm NaCl, pH 7.4) with an average apparent affinity, *K*_ITC_, of (8.06 ± 0.40) × 10^4^ m^−1^ (Fig. 2). Rabbit HRG has been frequently used in biochemical studies, owing to its higher abundance in rabbit plasma (˜ 0.9 mg mL^−1^) [41], but it possesses a longer HRR (Fig. S1). Examination of Zn^2+^ binding to serum-purified rabbit HRG with the same method and conditions revealed that the rabbit protein bound 10 mol eq. (*N* = 10.4) of Zn^2+^, similarly to the human HRG–Zn^2+^ interaction and in keeping with previously reported data [41], but with a lower average affinity than human HRG, with a *K*_ITC_ of (4.39 ± 0.33) × 10^4^ m^−1^ (Fig. S2).

**Fig 3:**
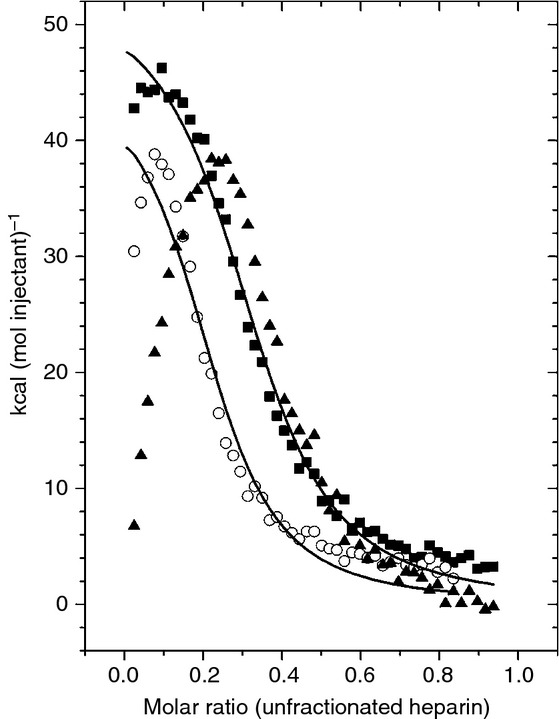
Isothermal titration calorimetry data showing the effect of Zn^2+^ on heparin binding to human histidine-rich glycoprotein (HRG). In the absence of Zn^2+^, the interaction between heparin and HRG for the first few injections gives rise to a less endothermic (or exothermic) component of the isotherm corresponding to a different overlapping mode of binding, which is increasingly pronounced in the presence of Zn^2+^. Here, 45 injections of 5 μL of 50 μm heparin (average molecular mass assumed to be 15 kDa) were delivered to samples of HRG (10 μm in buffer containing 50 mm Tris, 50 mm NaCl and 0 μm [], 1 μm [○] and 5 μm [] ZnCl_2_, pH 7.4) over a period of 10 s with an adequate interval (240 s) between injections. Zn^2+^ was included in the buffer at concentrations of 0, 1 and 5 μm. Raw data are shown in Figs. S6–S8.

The influence of Zn^2+^ on the heparin-binding properties of human HRG was probed with ITC. Unfractionated porcine plasma heparin (molecular mass range of 3–30 kDa) was titrated into samples of human HRG containing different concentrations of ZnCl_2_ (Fig. 3). The presence of Zn^2+^ had a marked effect on the mechanism by which human HRG bound heparin. In the absence of Zn^2+^, the interaction between heparin and HRG for the first few injections gave rise to a less endothermic (or exothermic) component of the isotherm. This initial form of heparin binding was more pronounced in the presence of 5 μm Zn^2+^. This reveals that heparin binds HRG via different ‘modes’, whereby the less endothermic or exothermic mode of binding occurs with higher affinity than the more endothermic, lower-affinity mode, and is modulated by Zn^2+^. The possibility that the isotherm reflects both Zn^2+^–heparin and heparin–HRG interactions was ruled out because the Zn^2+^ concentration was identical in both protein-containing and injectant solutions. It was possible to fit curves to the endothermic data collected in the absence and presence of 1 μm Zn^2+^, but not to the isotherm observed at 5 μm. The resultant curves suggest that the second, endothermic mode most probably corresponds to a single heparin site (*N* < 0.4 in each case). The calculated *K*_ITC_ values for this mode were (2.44 ± 0.25) × 10^6^ m^−1^ with no Zn^2+^ and (2.44 ± 0.54) × 10^6^ m^−1^ in the presence of 1 μm Zn^2+^, indicating that there is no Zn^2+^ dependence for this mode of binding. It is important to note, however, that the ‘real’ affinities are probably higher, as this analysis does not take into account binding via the first mode. It was also observed that there was a difference in the stoichiometry of heparin binding to HRG in the presence of 1 μm Zn^2+^ (as illustrated by a shift in the curve to the left) as compared with the data without Zn^2+^ or with 5 μm Zn^2+^. This correlates with a previous study revealing that complexes of 1 : 1 and 2 : 1 (HRG/heparin) can form, with formation of the 2 : 1 complex being enhanced by the presence of Zn^2+^ [42]. As unfractionated heparin was used in this instance, it was not possible to assign an accurate molecular mass to the titrant solution (an average mass of 15 kDa was used), and so the *x*-axis in Fig. 3 is, to a large degree, arbitrary. However, if we use the molar ratio of 0.4 observed in these experiments to represent the 1 : 1 complex (which is the calculated *N*-value for both the Zn^2+^-free and 5 μm Zn^2+^ datasets), then the molar ratio of 0.2 (which is the calculated *N*-value for the 1 μm Zn^2+^ dataset) can be taken to represent the 2 : 1 complex. The data here suggest that higher concentrations of Zn^2+^ (5 μm) inhibit formation of the 2 : 1 complex. The complexity of the interaction is a corollary of the molecules involved, as HRG is probably able to bind heparin at different regions, and heparin molecules themselves are heterogeneous (existing in varying chain lengths), and, in the presence of Zn^2+^, interact differently with HRG, depending on length.

**Fig 4:**
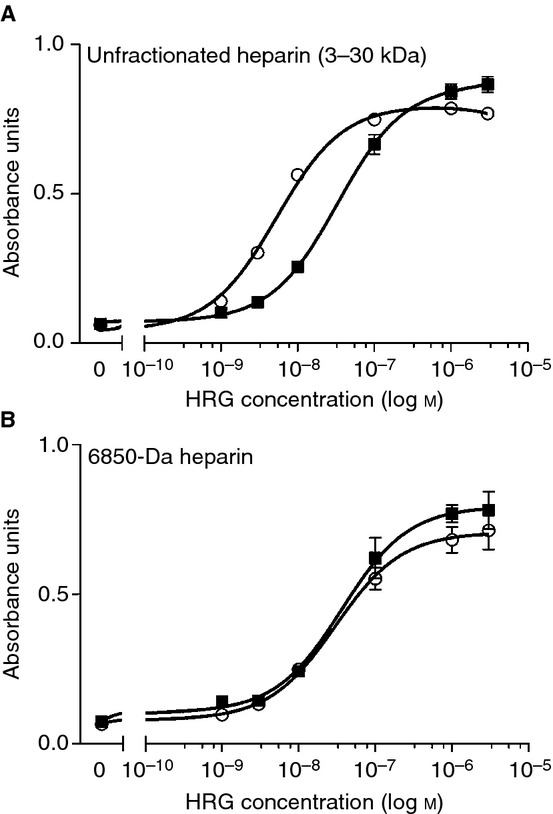
Analysis of histidine-rich glycoprotein (HRG)-heparin binding using an ELISA-based assay. Influence of Zn^2+^ on binding of human HRG to (A) unfractionated heparin (3–30 kDa) and (B) low molecular weight heparin (6850 Da). Heparin (25 μg mL^−1^) was coated overnight onto a heparin-binding plate in 50 mm HEPES, 150 mm NaCl, and 0.2% Tween-20 (pH 7.4). The plate was then washed with the same buffer, and then blocked with the same buffer containing 0.2% gelatin. Human HRG was then added over a range of concentrations (0–3 μm) with either 0 μm () or 1 μm (○) ZnCl_2_ in triplicate, and incubated for 2 h. Detection was performed with primary rabbit anti-HRG followed by alkaline phosphatase-linked anti-rabbit antibody, and observed with a *p*-nitrophenol phosphate substrate at 405 nm.

As it was problematic to obtain quantitative information from the ITC data, owing to the mix of interactions giving rise to the different enthalpies observed, an ELISA protocol was established to calculate the affinities involved in this interaction. Unfractionated (3–30 kDa) and fractionated LMWH (6850 Da) were used separately in these studies. In each case, HRG bound heparin in a concentration-specific manner (Fig. 4A,B). In the absence of Zn^2+^, the average apparent *K*_d_′ value was 32.9 nm (corresponding to *K*′ = 3.04 × 10^7^ m^−1^). This is considerably stronger than the affinity derived from the ITC data (*K*_ITC_ = 2.44 × 10^6^ m^−1^), and probably reflects the Zn^2+^-dependent mode of binding that could not be quantified by ITC. The affinity of HRG for the unfractionated heparin was even higher in the presence of 1 μm Zn^2+^ (average apparent *K*_d_^′^ = 5.1 nm). The stochiometry of binding was similar in both cases, suggesting that the two binding modes observed in the ITC experiments are mutually exclusive (i.e. coordination of Zn^2+^ does not create additional heparin-binding sites). These data suggest that even relatively small changes in plasma Zn^2+^ speciation are likely to affect the heparin-binding properties of HRG and its hemostatic functions. Zn^2+^ did not influence the ability of HRG to bind LMWH; average *K*_d_ values were ˜ 30 nm in both the presence and absence of 1 μm Zn^2+^ (Fig. 4B). Antithrombin has a high affinity for heparin, with a *K*_d_ in the region of 10–20 nm [43], and was reported to bind a fraction of heparin (termed low-affinity heparin) with a *K*_d_ of 19 μm [44]. Taking these numbers into account with the data obtained here, it is apparent that HRG is a stronger competitor for heparin in the presence of Zn^2+^.

Previous studies have indicated that the N1/N2 region and the HRRs of HRG interact with heparin, and that binding to the HRR is Zn^2+^-dependent [11,45]. From the data presented, it would appear that HRG binds heparins of essentially all chain lengths via its N1/N2 domain in a Zn^2+^-independent manner, forming a 1 : 1 complex. When larger heparin chains are present, binding affinity for HRG is enhanced by Zn^2+^. In addition, the data suggest that addition of 1 μm Zn^2+^ allows formation of 2 : 1 (HRG/heparin) complexes. This effect has previously been shown only to occur with heparins of ≥ 10 kDa [42]. Longer-chain heparins presumably offer greater potential for simultaneous binding of multiple HRG molecules to a single chain. However, this is stated with caution, as the data here do not fully reveal the binding mechanism. It is also unclear why addition of 5 μm Zn^2+^ averted formation of 2 : 1 complexes, but it is likely that a higher proportion of Zn^2+^ bound at the HRR would enhance heparin binding at this site, which would increase the number of heparin molecules bound per HRG molecule.

The data presented are significant, as heparins are used clinically as anticoagulants, although there are some complications with their use (particularly for unfractionated heparin). Unfractionated heparin is plagued by a narrow therapeutic window and an unpredictable dose–response profile, as well as other problems, including the inability to promote inhibition of fibrin-bound thrombin and platelet-bound factor Xa and the potential to trigger heparin-induced thrombocytopenia. LMWHs have a more predictable dose–response profile, but are still unable to inhibit fibrin-bound thrombin and platelet-bound factor Xa [46,47]. The observation that Zn^2+^ increases the affinity of HRG for unfractionated heparin (which contains heparins up to 30 kDa) and not LMWH may help to explain the clinical differences observed between the former and the latter.

**Fig 5:**
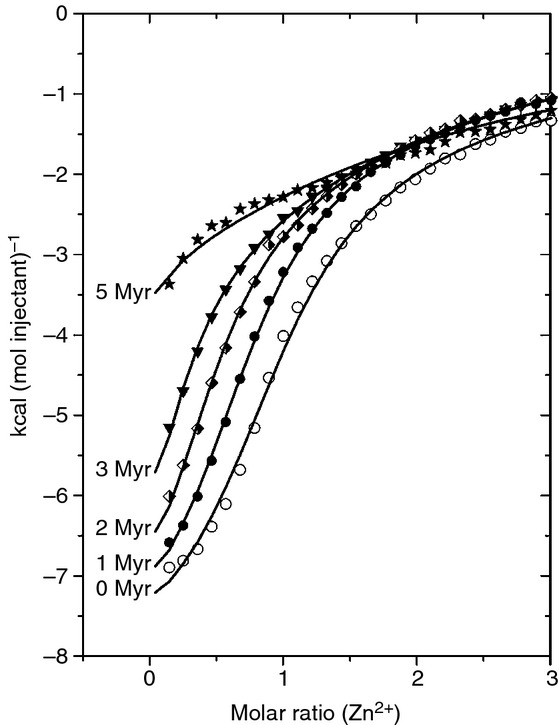
Isothermal titration calorimetry data showing the interaction between human serum albumin (HSA) and Zn^2+^ in the presence of 0 mol eq. (○), 1 mol eq. (•), 2 mol eq. (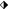), 3 mol eq. (▾) and 5 mol eq. (⋆) of myristate (Myr). HSA (50 μm) was incubated with the desired amount of Myr for 2 h at 37 °C. The HSA sample was then titrated with 5-μL injections of a 1.5 mm ZnCl_2_ solution (55 injections). The total Zn^2+^ concentration at the end of the experiment was 246 μm. Experiments were conducted in buffer containing 50 mm Tris and 140 mm NaCl (pH 7.4). For clarity, only the first halves of the curves are shown (see Fig. S3 for full dataset). The fits correspond to a ‘two-sets-of-sites’ model with the stoichiometric factor for the secondary binding site fixed at 1.00. Raw data are shown in Figs. S9–S14.

**Fig 6:**
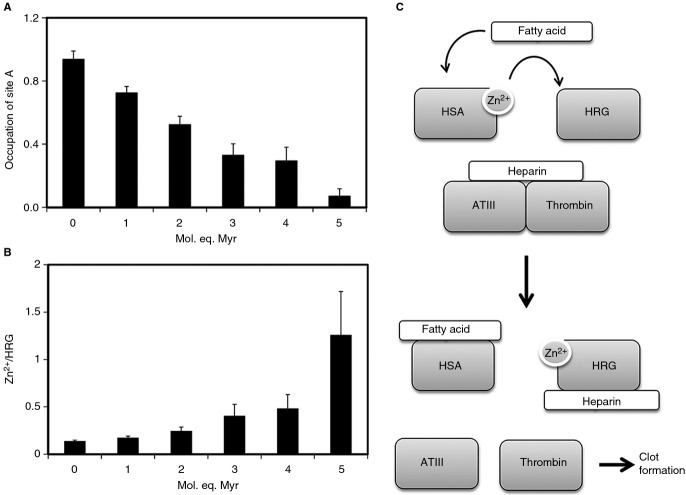
Speciation modeling analysis of plasma Zn^2+^. (A) Occupation of human serum albumin (HSA) site A in the presence of 0–5 mol. eq. myristate. (B) Proportion of Zn^2+^ bound to HRG in the presence of 0–5 mol. eq. myristate. Values for the stoichiometric factor for site A were extracted from several fits (Table S2) and averaged. For the determination of HRG loading with Zn^2+^ the conditional constants from Table 1 were used to model the distribution of Zn^2+^ (15 μm) in the presence of HSA (620 μm) and HRG (1 or 2 μm). Full speciation data are reported in Table S3. Errors correspond in each case to 1*σ*. (C) Reaction scheme showing the proposed effects that elevated FFA levels would have in relation to modulation of HRG–heparin interactions and destabilization of the thrombin-antithrombin III (ATIII) complex. Myr, myristate.

Recently, we examined the binding of Myr (C14) to bovine serum albumin (BSA) by using ITC. This revealed that even the presence of 1 mol eq. of Myr perturbed albumin's ability to bind Zn^2+^, and that 4 mol eq. of Myr was sufficient to almost completely suppress Zn^2+^ binding [29]. To examine the effect of FFAs on the Zn^2+^-binding properties of HSA, ITC was performed with HSA (50 μm), loaded with increasing molar equivalents of Myr (0–250 μm, corresponding to 0–5 mol eq.) prior to titration with ZnCl_2_ (1.5 mm). The resulting isotherms are shown in Fig. 5 (the full dataset is shown in Fig. S3), where trends for decreasing stoichiometry and a lowering of the overall affinity of HSA for Zn^2+^ are observed. Two classes of binding site were discernible for FFA-free HSA, yielding *K1*_ITC_ = 1.35 × 10^5^ m^−1^ and *K2*_ITC_ = 2.86 × 10^3^ m^−1^. The weaker-affinity binding site class corresponds to at least one further metal-binding site with non-negligible affinity for Zn^2+^; the existence of such secondary sites is well documented in the literature [48–51]. All fits shown in Fig. 5 correspond to a two-sets-of-sites model with *K1*_ITC_, the binding constant for the highest-affinity site (site A), now fixed at 1.35 × 10^5^ m^−1^, and the stoichiometric factor *N1* being varied. Various fitting approaches were explored (Tables S1 and S2), and, under all scenarios, the stoichiometric factor *N1* for site A decreased progressively, from 0.98 to 0.86 in the absence of Myr, to 0.01–0.13 in the presence of 5 mol eq. of Myr (Fig. 6A). From these data, it is clear that the high-affinity Zn^2+^ site had all but disappeared at 5 mol eq. of Myr, although some weak Zn^2+^-binding capacity (*K2*_ITC_ < 10^4^ m^−1^) from the secondary site(s) remained (Table S1). In contrast to what was observed for BSA [29], the secondary binding sites on HSA were not adversely affected by the presence of Myr. Overall, it may be concluded that even normal (˜ 1 mol eq.) FFA levels modulate the Zn^2+^-binding capacity of HSA, but that pathologic levels (up to 5 mol eq. [31–36]) severely affect or nearly abolish high-affinity Zn^2+^ binding. It is likely that the physiologically pertinent longer-chain fatty acids (C16 and C18), owing to their higher affinity for HSA [40], have an at least similar if not more pronounced effect.

With the Zn^2+^-binding constant data for HRG and for HSA in the presence and absence of FFA in hand, it was possible to explore whether an increase in plasma FFA levels is likely to lead to Zn^2+^ redistribution from HSA to HRG. All *K*_ITC_ values were corrected for competition with Tris [29], but, as all experiments were carried out at physiologic pH and ionic strength, the resulting conditional constants (Table 1) are otherwise valid for the conditions in plasma. Using these constants, we modeled Zn^2+^ speciation in the HRG–HSA–FFA system on the basis of typical physiologic concentrations of exchangeable Zn^2+^ (15 μm), HSA (620 μm), and HRG (1 or 2 μm). The effect of FFA was determined as a reduction in the availability of site A, with the numbers for *N1* determined above. Weaker binding to the secondary sites on HSA was also taken into account, with various combinations of *N2* and *K2* derived from the fits (Table S2). All calculated speciation values are reported in Table S3, and the most salient findings are shown in Fig. 6. As the availability of site A decreased (Fig. 6A), some Zn^2+^ became unbound, some Zn^2+^ became bound by the secondary site(s) on albumin, and a significant proportion became bound to HRG (Fig. 6B). At the highest Myr level, HRG had ˜ 1.25 mol eq. of Zn^2+^ bound, as compared with ˜ 0.15–0.25 mol eq. at physiologically normal FFA levels (1–2 mol eq.). According to the ELISA assay data shown in Fig. 4, an equimolar amount of Zn^2+^ is sufficient to significantly increase the affinity of HRG for unfractionated heparin. It needs to be emphasized that our estimates are deliberately conservative, and that a reduction in site A availability to 0.07 still corresponds to ˜ 40 μm – in principle, still more than enough to bind all exchangeable Zn^2+^. Nevertheless, the binding constants and concentrations of HSA and HRG seem to be so finely balanced that even partial obliteration of site A on HSA leads to a notable shift of Zn^2+^ from HSA to HRG. The formation of up to 0.9 μm ‘free Zn^2+^’ is also interesting (Fig. S4); it is possible that this fraction becomes more available for interaction with other plasma proteins and/or for cellular uptake via ZIP transporters by endothelial or other cells. Significantly, a reduction in total plasma Zn^2+^ is also a hallmark of several disease states that are characterized by high plasma FFA levels [52–56].

**Table 1:** Conditional Zn^2+^ binding constants for human serum albumin (HSA) and histidine-rich glycoprotein (HRG)

Protein	Fixed parameter	*K*_ITC_	*K*′
HRG	–	(8.06 ± 0.40) × 10^4^	1.63 × 10^5^
HSA (site A)	–	(1.35 ± 0.20) × 10^5^	2.73 × 10^5^
HSA [secondary site(s)]	*N2* = 1	(6.1 ± 1.5) × 10^3^	1.2 × 10^4^
	*N2* = 2	(7.0 ± 2.7) × 10^3^	1.4 × 10^4^

The final conditional constants *K*′ valid for pH 7.4 and physiologic ionic strength were derived from the *K*_ITC_ constants by correcting for competition with 50 mm Tris [29]. *K*_ITC_ for site A was derived from fitting the data in the absence of myristate (Myr) to a sequential binding sites model with two sites (Table S1). In the case of the secondary site(s) on HSA, the averages from fitting data in the presence and absence of Myr are reported

Speciation modeling of plasma Zn^2+^ based on the presented data suggests that elevated FFA levels (as observed in certain pathologic conditions) will modulate HRG–heparin interactions, which could potentially impact on coagulation (Fig. 6C). Our model only considered serum albumin and HRG, and did not take into account other Zn^2+^-binding molecules present in the circulation that could bind at least some of the Zn^2+^ displaced from albumin. However, the abundance of HRG in plasma (micromolar levels) and its affinity for Zn^2+^ suggest that it would probably bind a significant proportion of displaced Zn^2+^. Furthermore, both ITC (qualitatively) and ELISA assay data (quantitatively) indicated that only a small proportion of the Zn^2+^ displaced from serum albumin (1–2 μm) is required to have a pronounced effect on the affinity of HRG for heparin.

In addition to heparin neutralization, HRG binds with high affinity to plasminogen in a Zn^2+^-dependent manner [57]. Despite this, the effects of this interaction on plasminogen conversion to plasmin or its fibrinolytic activity remain unknown. Moreover, HRG is known to interact with fibrinogen and compete with thrombin binding on the γ-chain of the protein [58]. This interaction is also Zn^2+^-dependent, and its effects on fibrin clot formation or structure have not been studied. This means that hyperactivation of HRG in disease states may also influence hemostatic functioning through other mechanisms. Zn^2+^ is also known to influence thrombosis and hemostasis through interaction with other proteins. For example, Zn^2+^ may promote platelet aggregation by enhancing the interactions of fibrinogen with its cognate receptor, α_IIb_β_3_ [59], and of high molecular weight kininogen and FXII with platelet glycoprotein Ib [60], at the platelet surface. Thus, the full impact of FFA-mediated displacement of Zn^2+^ from HSA on hemostasis may not be limited to the modulation of HRG–heparin interactions.

The results of the current study are therefore compelling, and provide evidence to suggest that Zn^2+^-dependent formation of HRG–heparin complexes, following FFA binding to serum albumin, constitutes a novel molecular mechanism for the development of hemostatic complications in individuals with high plasma FFA levels. Thus, maintenance and monitoring of plasma FFA levels may prove useful in preventing thrombosis and the formation of obstructive clots.

## Addendum

O. Kassaar, U. Schwarz-Linek, C. A. Blindauer, and A. J. Stewart study concept and design. O. Kassaar acquisition of data. O. Kassaar, U. Schwarz-Linek, C. A. Blindauer, and A. J. Stewart analysis and interpretation of data. O. Kassaar, U. Schwarz-Linek, C. A. Blindauer, and A. J. Stewart drafting of the manuscript. All authors critically reviewed the manuscript and approved the final version.

## Supporting Information

Fig S1 Alignment of human and rabbit HRG amino acid sequences. **Fig. S2.** Full ITC data (including raw data) for Zn^2+^ binding to rabbit HRG. **Fig. S3.** ITC data showing the interaction between HSA and Zn^2+^ in the presence of 0–5 mol eq. of myristate. **Fig. S4.** Predicted unbound Zn^2+^ concentrations in the presence of 0–5 mol eq. of myristate. **Fig. S5.** Full ITC data (including raw data) for Zn^2+^ binding to human HRG. **Fig. S6.** Full ITC data (including raw data) for heparin binding to human HRG in the absence of Zn^2+^. **Fig. S7.** Full ITC data (including raw data) for heparin binding to human HRG in the presence of 1 µm Zn^2+^. **Fig. S8.** Full ITC data (including raw data) for heparin binding to human HRG in the presence of 5 µm Zn^2+^. **Fig. S9.** Full ITC data (including raw data) for Zn^2+^ binding to human HRG in the absence of myristate. **Fig. S10.** Full ITC data (including raw data) for Zn^2+^ binding to human HRG in the presence of 1 mol eq. of myristate. **Fig. S11.** Full ITC data (including raw data) for Zn^2+^ binding to human HRG in the presence of 2 mol eq. of myristate. **Fig. S12.** Full ITC data (including raw data) for Zn^2+^ binding to human HRG in the presence of 3 mol eq. of myristate. **Fig. S13.** Full ITC data (including raw data) for Zn^2+^ binding to human HRG in the presence of 4 mol eq. of myristate. **Fig. S14.** Full ITC data (including raw data) for Zn^2+^ binding to human HRG in the presence of 5 mol eq. of myristate. **Table S1.** ITC data fitting approaches for ITC experiments examining Zn^2+^ binding in the presence of 0–5 mol eq. of myristate. **Table S2.** Fitting results for ITC experiments examining Zn^2+^ binding in the presence of 0–5 mol eq. of myristate. **Table S3.** Results from Zn^2+^ speciation modeling.
