# Supplementary material for: Plasma free fatty acid levels influence Zn2+-dependent histidine-rich glycoprotein–heparin interactions via an allosteric switch on serum albumin
Source: J Thromb Haemost. 2014 Nov 22;13(1):101–10. doi: 10.1111/jth.12771 (PMC4309485; doi:10.1111/jth.12771)
Supplement: Fig S1 — Alignment of human and rabbit HRG amino acid sequences. Fig. S2. Full ITC data (including raw data) for Zn2+ binding to rabbit HRG. Fig. S3. ITC data showing the interaction between HSA and Zn2+ in the presence of 0–5 mol eq. of myristate. Fig. S4. Predicted unbound Zn2+ concentrations in the presence of 0–5 mol eq. of myristate. Fig. S5. Full ITC data (including raw data) for Zn2+ binding to human HRG. Fig. S6. Full ITC data (including raw data) for heparin binding to human HRG in the absence of Zn2+. Fig. S7. Full ITC data (including raw data) for heparin binding to human HRG in the presence of 1 µm Zn2+. Fig. S8. Full ITC data (including raw data) for heparin binding to human HRG in the presence of 5 µm Zn2+. Fig. S9. Full ITC data (including raw data) for Zn2+ binding to human HRG in the absence of myristate. Fig. S10. Full ITC data (including raw data) for Zn2+ binding to human HRG in the presence of 1 mol eq. of myristate. Fig. S11. Full ITC data (including raw data) for Zn2+ binding to human HRG in the presence of 2 mol eq. of myristate. Fig. S12. Full ITC data (including raw data) for Zn2+ binding to human HRG in the presence of 3 mol eq. of myristate. Fig. S13. Full ITC data (including raw data) for Zn2+ binding to human HRG in the presence of 4 mol eq. of myristate. Fig. S14. Full ITC data (including raw data) for Zn2+ binding to human HRG in the presence of 5 mol eq. of myristate. Table S1. ITC data fitting approaches for ITC experiments examining Zn2+ binding in the presence of 0–5 mol eq. of myristate. Table S2. Fitting results for ITC experiments examining Zn2+ binding in the presence of 0–5 mol eq. of myristate. Table S3. Results from Zn2+ speciation modeling. [file jth0013-0101-sd1.docx]

**Supplementary Information**

Plasma free fatty acid levels influence Zn^2+^-dependent histidine-rich glycoprotein-heparin interactions via an allosteric switch on serum albumin

Omar Kassaar, Ulrich Schwarz-Linek, Claudia A. Blindauer, Alan J. Stewart

**Figure S1: Alignment of human and rabbit HRG amino acid sequences.**

**Figure S2: Full ITC data (including raw data) for Zn^2+^ binding to rabbit HRG.**

**Figure S3: ITC data showing the interaction between HSA and Zn^2+^ in the presence of 0-5 mol. eq. of myristate.**

**Figure S4: Predicted unbound Zn^2+^ concentrations in the presence of 0-5 mol. eq. of myristate.**

**Table S1: ITC data fitting approaches for ITC experiments examining Zn^2+^-binding in the presence of 0-5 mol. eq. of myristate.**

**Table S2:** **Fitting results for ITC experiments examining Zn^2+^-binding in the presence of 0-5 mol. eq. of myristate.**

**Table S3: Results from Zn^2+^ speciation modelling.**

**Figure S5: Full ITC data (including raw data) for Zn^2+^ binding to human HRG.**

**Figure S6: Full ITC data (including raw data) for heparin binding to human HRG in the absence of Zn^2+^.**

**Figure S7: Full ITC data (including raw data) for heparin binding to human HRG in the presence of 1 µM Zn^2+^.**

**Figure S8: Full ITC data (including raw data) for heparin binding to human HRG in the presence of 5 µM Zn^2+^.**

**Figure S9: Full ITC data (including raw data) for Zn^2+^ binding to human HRG in the absence of myristate.**

**Figure S10: Full ITC data (including raw data) for Zn^2+^ binding to human HRG in the presence of 1 molar equivalent of myristate.**

**Figure S11: Full ITC data (including raw data) for Zn^2+^ binding to human HRG in the presence of 2 molar equivalents of myristate.**

**Figure S12: Full ITC data (including raw data) for Zn^2+^ binding to human HRG in the presence of 3 molar equivalents of myristate.**

**Figure S13: Full ITC data (including raw data) for Zn^2+^ binding to human HRG in the presence of 4 molar equivalents of myristate.**

**Figure S14: Full ITC data (including raw data) for Zn^2+^ binding to human HRG in the presence of 5 molar equivalents of myristate.**

Human HRG VSPTDCSAVEPEAEKALDLINKRRRDGYLFQLLRIADAHLDRVENTTVYYLVLDVQESDC

Rabbit HRG LTPTDCKTTKPLAEKALDLINKWRRDGYLFQLLRVADAHLDGAESATVYYLVLDVKETDC

::****.:.:* ********** ***********:****** .*.:*********:*:**

Human HRG SVLSRKYWNDCEPPDSRRPSEIVIGQCKVIATRHSHESQDLRVIDFNCTTSSVSSALANT

Rabbit HRG SVLSRKHWEDCDPDLTKRPSLDVIGQCKVIATRYSDEYQTLRLNDFNCTTSSVSSALANT

******:*:**:* ::*** ***********:*.* * **: ****************

Human HRG KDSPVLIDFFEDTERYRKQANKALEKYKEENDDFASFRVDRIERVARVRGGEGTGYFVDF

Rabbit HRG KDSPVLFDFIEDTEPFRKSADKALEVYKSESEAYASFRVDRVERVTRVKGGERTNYYVDF

******:**:**** :**.*:**** **.*.: :*******:***:**:*** *.*:***

Human HRG SVRNCPRHHFPRHPNVFGFCRADLFYDVEALDLESPKNLVINCEVFDPQEHENINGVPPH

Rabbit HRG SVRNCSRSHFHRHP-AFGFCRADLSFDVEASNLENPEDVIISCEVFNFEEHGNISGFRPH

*****.* ** *** .******** :**** :**.*::::*.****: :** **.*. **

Human HRG LGHPFHWGGHERSSTTKPPFKPHGSRDHHHPHKPHEHGPPPPPDERDHSHGPPLPQGPPP

Rabbit HRG LG--------------KTPLGTDGSRDHHHPHKPHKFGCPPPQEGEDFSEGPPLQGGTPP

** *.*: ..************:.* *** : .*.*.**** *.**

Human HRG LLPMSCSSCQHATFGTNGAQRHSHNNNSS----DLHPHKHHSHEQHPHGHHPHAHHPHEH

Rabbit HRG LSPPFRPRCRHRPFGTNETHRFPHHRISVNIIHRPPPHGHHPHGPPPHGHHPHGPPPHGH

* * . *:* .**** ::*..*:. * ** **.* *******. ** *

Human HRG DTHR-------------------QHPHGHHPHGHHPHGHHPHGHHPHGHHPHCHDFQDYG

Rabbit HRG PPHGPPPRHPPHGPPPHGHPPHGPPPHGHPPHGPPPHGHPPHGPPPHGHPPHGHGFHDHG

.* **** *** **** *** **** ** *.*:*:*

Human HRG PCDPPPHNQG---HCCHGHGPPPGHLRRRGPGKGPRPFHCRQIGSVYRLPPLRKGEVLPL

Rabbit HRG PCDPPSHKEGPQDLHQHAMGPPPKHPGKRGPGKGHFPFHWRRIGSVYQLPPLQKGEVLPL

*****.*::* *. **** * :****** *** *:*****:****:*******

Human HRG PEANFPSFPLPHHKHPLKPDNQPFPQSVSESCPGKFKSGFPQVSMFFTHTFPK

Rabbit HRG PEANFPQLLLRNHTHPLKPEIQPFPQVASERCPEEFNGEFAQLSKFFPSTFPK

******.: * :*.*****: ***** .** ** :*:. *.*:* **. ****

**Figure S1.** Alignment of human and rabbit HRG amino acid sequences. The histidine-rich region is shown in red. Alignment was performed using ClustalW available at the Swiss Institute of Bioinformatics webserver. “*“ fully-conserved residues; “:” conserved substitutions; “.” semi-conserved substitutions.

**Figure S2.** ITC data for Zn^2+^ binding to rabbit HRG. 55 injections of 5 μl of 150 μM ZnCl_2_ were delivered to samples of HRG (10 μM in buffer containing 50 mM Tris, 140 mM NaCl at pH 7.4) over 10 s with an adequate interval (240 s) between injections to allow complete equilibration.

**Figure S3.** ITC data showing the interaction between HSA and Zn^2+^ in the presence of 0 (○), 1(●), 2 (), 3 (
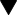
), 4 (
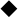
) and 5 (
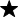
) mol. eq. of myristate. 50 µM HSA was incubated with the desired amount of myristate for 2 hours at 37 °C. The HSA sample was then titrated with 5 µl injections of a 1.5 mM ZnCl_2_ solution for 55 injections. Experiments were conducted in buffer containing 50 mM Tris, 140 mM NaCl, pH 7.4. The fits correspond to a two-sets-of-sites model with the stoichiometric factor for the secondary binding site fixed at 1.00.

**Figure S4.** Predicted unbound Zn^2+^ concentrations in the presence of 0-5 mol. eq. of myristate. This corresponds to the fraction of Zn^2+^ not bound to either HSA or HRG in the final speciation model.

**Table S1.** ITC data fitting approaches for ITC experiments examining Zn^2+^-binding in the presence of 0-5 mol. eq. of myristate. The values for K1’ and DH1 in fits 2-6 are derived from fit 1 (see Table S2). The values for K2’ and DH2 in fit 2 are also derived from fit 1. The respective values in fit 3 are derived from fit 5. All entries marked “v” signify parameters that were varied. Results for these varied parameters are given in Table S2.

| Fit | Model | Fixed parameters | | | | | |
| --- | --- | --- | --- | --- | --- | --- | --- |
|  |  | N1 | *K*1’ (x10^-5^) | ΔH1 (kcal/mol) | N2 | *K*2’ (x10^-3^) | ΔH2 (kcal/mol) |
| Fit 1 | 2 sequential binding sites | n.a. | v | v | n.a. | v | v |
|  |  |  |  |  |  |  |  |
| Fit 2 | Two sets of sites | v | 1.35 | -8245 | v | 2.86 | -13620 |
| Fit 3 | Two sets of sites | v | 1.35 | -8245 | v | 3.27 | -6679 |
| Fit 4 | Two sets of sites | v | 1.35 | -8245 | v | v | v |
| Fit 5 | Two sets of sites | v | 1.35 | -8245 | 2.00 | v | v |
| Fit 6 | Two sets of sites | v | 1.35 | -8245 | 1.00 | v | v |

**Table S2.** Fitting results for ITC experiments examining Zn^2+^-binding in the presence of 0-5 mol. eq. of myristate.

| Fitted Parameter | Fit | 0 Myr | 1 Myr | 2 Myr | 3 Myr | 4 Myr | 5 Myr |
| --- | --- | --- | --- | --- | --- | --- | --- |
| *K*1’ (x10^-5^) | Fit 1 | 1.35 |  |  |  |  |  |
| ΔH1 (kcal/mol) |  | -8245 |  |  |  |  |  |
| *K*2’ (x10^-3^) |  | 2.86 |  |  |  |  |  |
| ΔH2 (kcal/mol) |  | -13620 |  |  |  |  |  |
|  |  |  |  |  |  |  |  |
| N1 | Fit 2 | 0.945 | 0.736 | 0.547 | 0.372 | 0.348 | 0.093 |
|  | Fit 3 | 0.986 | 0.745 | 0.575 | 0.405 | 0.400 | 0.129 |
|  | Fit 4 | 0.862 | 0.674 | 0.487 | 0.302 | 0.267 | 0.083 |
|  | Fit 5 | 0.983 | 0.775 | 0.559 | 0.353 | 0.282 | 0.034 |
|  | Fit 6 | 0.911 | 0.694 | 0.449 | 0.220 | 0.173 | 0.014 |
|  |  |  |  |  |  |  |  |
| N2 | Fit 2 | 1.08 | 0.966 | 1.09 | 1.22 | 1.49 | 1.51 |
|  | Fit 3 | 1.98 | 1.61 | 2.04 | 2.30 | 2.81 | 2.93 |
|  | Fit 4 | 0.453 | 0.810 | 1.32 | 1.64 | 1.89 | 2.84 |
|  |  |  |  |  |  |  |  |
| *K*2’ (x 10^-3^) | Fit 4 | 4.20 | 5.35 | 6.84 | 9.44 | 9.21 | 12.50 |
|  | Fit 5 | 3.27 | 4.42 | 6.71 | 10.00 | 9.51 | 7.95 |
|  | Fit 6 | 4.06 | 5.27 | 6.75 | 8.28 | 7.04 | 5.25 |
|  |  |  |  |  |  |  |  |
| ΔH2 (kcal/mol) | Fit 4 | -27990 | -12390 | -7650 | -6202 | -6642 | -4054 |
|  | Fit 5 | -6679 | -5028 | -4806 | -4770 | -6154 | -6696 |
|  | Fit 6 | -12460 | -9924 | -10490 | -11260 | -14420 | -15400 |

From these data, it is clear that fit N^o^ 4 gives unreasonable trends for *K*2 and ΔH2. Fits No 2 and N^o^ 3 gave unacceptably high values for Χ^2^ for the data in presence of 2 - 5 Myr (between 13530 and 47620). Fits N^o^s 5 and 6 gave low Χ^2^ values for all six datasets, with those for fit N^o^ 6 (N2 set to 1.00) slightly lower in all but one cases.

**Table S3.** Results from Zn^2+^ speciation modelling. An HSA concentration of 620 µM and a Zn^2+^ concentration of 15 µM were used in all models. Log *K*1’, the conditional binding constant for site A, was set at 5.44; this is derived from *K*_ITC_ = 1.35 x 10^5^, corrected for competition with 50 mM Tris. Similarly, log *K’* for HRG was set at 5.22, derived from *K*_ITC_ = 8.06 × 10^4^ M^-1^.

| Model | Mol. eq. Myr | % Zn | | | | | | | |
| --- | --- | --- | --- | --- | --- | --- | --- | --- | --- |
|  |  | unbound | | HSA site A | | HRG | | HSA secondary site(s) | |
| N2 = 1; log *K*2’ = 4.09; HRG = 1 μM | 0 | 0.62 | 93.69 | | 0.99 | | 4.71 | |  |
|  | 1 | 0.80 | 91.83 | | 1.27 | | 6.10 | |  |
|  | 2 | 1.20 | 87.76 | | 1.89 | | 9.14 | |  |
|  | 3 | 2.25 | 77.23 | | 3.45 | | 17.07 | |  |
|  | 4 | 2.73 | 72.42 | | 4.15 | | 20.70 | |  |
|  | 5 | 8.61 | 15.18 | | 11.55 | | 64.66 | |  |
|  |  | | | |  | |  | |  |
| N2 = 1; log *K*2’ = 4.09;  HRG = 2 μM | 0 | 0.61 | 92.78 | | 1.95 | | 4.66 | |  |
|  | 1 | 0.79 | 90.68 | | 2.51 | | 6.02 | |  |
|  | 2 | 1.18 | 86.14 | | 3.72 | | 8.97 | |  |
|  | 3 | 2.17 | 74.70 | | 6.68 | | 16.46 | |  |
|  | 4 | 2.61 | 69.61 | | 7.96 | | 19.82 | |  |
|  | 5 | 7.65 | 13.90 | | 20.92 | | 57.54 | |  |
|  |  | | | |  | |  | |  |
| N2 = 2; log *K*2’ = 4.15; HRG = 1 μM | 0 | 0.54 | 89.09 | | 0.87 | | 9.49 | |  |
|  | 1 | 0.67 | 86.51 | | 1.07 | | 11.75 | |  |
|  | 2 | 0.89 | 82.10 | | 1.42 | | 15.59 | |  |
|  | 3 | 1.29 | 74.06 | | 2.04 | | 22.61 | |  |
|  | 4 | 1.53 | 69.34 | | 2.39 | | 26.73 | |  |
|  | 5 | 4.03 | 20.05 | | 5.95 | | 69.97 | |  |
|  |  | |  | |  | |  | |  |
| N2 = 2; log *K*2’ = 4.15;  HRG = 2 μM | 0 | 0.54 | 88.33 | | 1.72 | | 9.41 | |  |
|  | 1 | 0.66 | 85.59 | | 2.12 | | 11.62 | |  |
|  | 2 | 0.88 | 80.96 | | 2.79 | | 15.37 | |  |
|  | 3 | 1.27 | 72.60 | | 3.99 | | 22.14 | |  |
|  | 4 | 1.49 | 67.75 | | 4.68 | | 26.08 | |  |
|  | 5 | 3.79 | 19.04 | | 11.26 | | 65.91 | | |

**Figure S5.** Full ITC data (including raw data) for Zn^2+^ binding to human HRG. corresponding to data shown in Figure 2.

**Figure S6:** Full ITC data (including raw data) for heparin binding to human HRG in the absence of Zn^2+^, corresponding to data shown in Figure 3.

**Figure S7:** Full ITC data (including raw data) for heparin binding to human HRG in the presence of 1 µM Zn^2+^, corresponding to data shown in Figure 3.

**Figure S8:** Full ITC data (including raw data) for heparin binding to human HRG in the presence of 5 µM Zn^2+^, corresponding to data shown in Figure 3.

**Figure S9:** Full ITC data (including raw data) for Zn^2+^ binding to human HRG in the absence of myristate, corresponding to data shown in Figure 5.

**Figure S10:** Full ITC data (including raw data) for Zn^2+^ binding to human HRG in the presence of 1 molar equivalent of myristate, corresponding to data shown in Figure 5.

**Figure S11:** Full ITC data (including raw data) for Zn^2+^ binding to human HRG in the presence of 2 molar equivalents of myristate, corresponding to data shown in Figure 5.

**Figure S12:** Full ITC data (including raw data) for Zn^2+^ binding to human HRG in the presence of 3 molar equivalents of myristate, corresponding to data shown in Figure 5.

**Figure S13:** Full ITC data (including raw data) for Zn^2+^ binding to human HRG in the presence of 4 molar equivalents of myristate, corresponding to data shown in Figure 5.

**Figure S14:** Full ITC data (including raw data) for Zn^2+^ binding to human HRG in the presence of 5 molar equivalents of myristate, corresponding to data shown in Figure 5.
